# A survey of Australian and New Zealand medical parents' experiences of infertility, pregnancy, and parenthood

**DOI:** 10.3389/fmed.2022.943112

**Published:** 2022-07-27

**Authors:** Jasmina Kevric, Katherine Suter, Russell Hodgson, Grace Chew

**Affiliations:** ^1^Division of Surgery, Northern Health, Epping, VIC, Australia; ^2^Department of Surgery, University of Melbourne, Austin Health, Heidelberg, VIC, Australia; ^3^Department of Surgery, Western Health, Melbourne, VIC, Australia; ^4^Department of Surgery, University of Melbourne, Epping, VIC, Australia; ^5^Breast Screen Victoria, St Vincent's Hospital, Melbourne, VIC, Australia

**Keywords:** pregnancy, surgeons, breastfeeding, complication, infertility

## Abstract

**Objective:**

To describe the incidence of infertility, pregnancy complications, and breastfeeding practices among Australian and New Zealand doctors and identify factors associated with increased pregnancy complication rates.

**Methods:**

A survey of ANZ doctors using an online questionnaire during November 2021.

**Results:**

One thousand ninety-nine completed responses were received. The median age of female doctors at the time of their first child was 32.4. Fertility testing was undertaken by 37%, with 27% having *in vitro* fertilization. More than 60% of respondents delayed family planning due to work. Pregnancy loss occurred in 36% of respondents, and 50% suffered a pregnancy complication. There were significant differences between specialists, with surgeons working longer hours before and after pregnancy, but having greater access to maternity leave than general practitioners.

**Conclusion:**

Female doctors delay starting and completing their family due to work-related demands and structural biases in career progression, which may result in higher infertility and pregnancy complication rates.

## Introduction

The number of female doctors in Australia's and New Zealand's workforce is on the rise (1). Australia's national medical board AHPRA reported that in 2021 44.8% of the registered medical professionals are female compared with only 38.6% in 2012 (2). This trend has followed within speciality practice, with figures showing 40.2% of specialists are women, up from only 35.7% 5 years ago (2). Some specialties that are starting from a low base (male dominated specialties) are also increasing, with The Royal Australasian College of Surgeons 2018 census confirming the increasing numbers of women in surgery from 9 to 12% (3).

Despite these advances in gender equality, combining parenthood and medicine has proven challenging for many female doctors. The current medical training structure is centered around the prime reproductive years of women. Systemic barriers in medicine and strong paternalistic culture have resulted in many doctors delaying pregnancy until after training. However, the longer women choose to postpone starting a family, the rate of complications rises considerably. This is particularly apparent after 35 years old, where there is a significantly higher risk of infertility, pregnancy loss, fertility interventions, and adverse neonatal outcomes (4). Work-related barriers such as working longer than 42 h per week or long and irregular shifts have shown similar outcomes (5). Further issues such as minimal parental leave, poor peer support and scarce breastfeeding spaces may also create many obstacles for female doctors to balance parenthood and their careers.

A recent JAMA article demonstrated a high infertility rate among American female surgeons, more than twice the rate of the general population (6). The authors called for urgent structural reform to support both men and women as our culture continues to prolong pregnancy. Although many studies across the globe have examined these important issues with infertility and motherhood for decades, there is a paucity of data within Australia. This research aims to describe the incidence of infertility, pregnancy complications, and breastfeeding practices among Australian and New Zealand doctors and identify factors associated with increased pregnancy complication rates.

## Methods

To address the aim of the study, a survey methodology was employed as a more thorough prospective generational study of female doctors was not ethically or practically feasible. Social media was chosen for survey distribution due to the heterogeneity of requirements by sub-specialty colleges for approval and distribution of such a survey. Ethical approval was obtained from the Northern Health Ethics and Governance Committee (HREC 79540).

### Definitions

For the purposes of this paper, the terms female and male refer to the gender of the participant, as recorded by the participant themselves. Sex was not recorded. Parenthood was defined as any participant identifying themselves as a parent, regardless of gender.

### Participants

An invitation to participate in a survey to assess experiences with fertility, pregnancy complications, and breastfeeding practices at work was posted on the Medical Parents Facebook Page containing over 11,000 AHPRA registered doctors although it is impossible to know how many of these members were active members (people who viewed the page within the past 3 months). Members of this Facebook group are required to confirm AHPRA registration to join this group, however no other demographic data (including location and employment details) are collected. Inclusion criteria consisted of all female doctors who had attempted and/or succeeded in becoming pregnant and who completed the survey, and all male doctors with a non-doctor female partner who had attempted and/or succeeded in becoming pregnant, and who completed the survey. Completion of the survey was defined as completing at least the gender question and 25% of the relevant questions. The data collection lasted for 4 weeks; with a reminder posted at the 2-week mark.

### Questionnaire

The questionnaire was a combination of 2 established and externally validated surveys: the recent US survey comprising a number of questions addressing fertility, pregnancy experiences and working conditions (6), and the Indiana University School of Medicine survey exploring breastfeeding practices among physicians (7). The REDCap system was used to distribute and store responses (8). Demographic information such as age, gender, relationship status and ethnicity was collected as well as the specialty of the participants and age of the first pregnancy. All data received were anonymous.

### Statistical analysis

Statistical analysis was performed using SPSS (IBM, Armonk, USA). Comparisons of categorical data were compared using Kruskal-Wallis, Chi-Square or Fisher's Exact Test. Continuous data was assessed for normality using the Shapiro-Wilk test for normality, with an alpha of >0.05 used to define normality. Student's *t*-tests or Mann-Whitney (rank sum) tests were used to test for differences in normally and non-normally distributed variables, respectively, with one-way ANOVA used for multiple groups. Statistical significance was set at a *p*-value of 0.05 for all analyses.

## Results

One thousand ninety-nine complete responses were received from over 11,000 members of the Medical Parents Facebook Page over the accrual period, of these 1,040 were from female doctors, 44 were from male doctors, and 15 did not disclose their gender. As it is not possible to know how many members of the group were active members, it is not possible to ascertain the response rate. The majority of respondents were married or in a domestic partnership, and of Caucasian ethnicity with a mix of specialty interests.

Comparison between female and male participants was performed to assess potential differences between medical and non-medical females. Although the age of participants (and their female partners) was similar, male doctors and their partners were both younger than female doctors at the time of their first child's birth (Table 1). Male doctors were more likely to have 3 children (25.6%) compared to female doctors (12.8%). The partners of male doctors worked less hours and a higher proportion did not return to work after giving birth. No differences were seen in pregnancy outcomes, although this may have been due to the low number of male participants recruited. A large number of female doctors reported to have undergone fertility testing (37.0%), and one in four female doctors required assisted reproductive therapy (ART). Pregnancy loss occurred in 36.4% of female doctors. Of particular note, the rate of pregnancy complications amongst female doctors was 49.9%.

**Table 1:** Comparison between female and male participants.

	**Female (*****n*** = **1,040)g**	**Male (*****n*** = **44)**	***p*** **-value**
Age participant (mean ± sd)	36.9 ± 5.4	36.9 ± 5.3	0.991
Age of female partner	–	36.5 ± 5.2	
Specialty	(*n* = 1,039)	(*n* = 44)	<0.001
Surgery	154 (14.8%)	17 (38.6%)	
Medicine	169 (16.3%)	9 (20.5%)	
General practice	287 (27.6%)	6 (13.6%)	
Pediatrics	94 (9.0%)	0	
Radiology	7 (0.7%)	0	
Other	328 (31.6%)	12 (27.3%)	
Age at first child's birth (median, IQR)	32 (30, 34)	31 (29, 33)	0.018
Age of female partner at first child's birth	–	31 (29, 32)	<0.001^*^
How many biological children?	(*n* = 970)	(*n* = 39)	0.039
1	419 (43.2%)	10 (25.6%)	
2	400 (41.2%)	17 (43.6%)	
3	124 (12.8%)	10 (25.6%)	
4	22 (2.3%)	1 (2.6%)	
>4	5 (0.5%)	1 (2.6%)	
Do you have the number of children you want for your family?	(*n* = 971)	(*n* = 39)	0.103
Yes	441 (45.4%)	23 (59.0%)	
Have you had fertility testing?	(*n* = 1,038)		0.205
Yes	384 (37.0%)	12 (27.3%)	
Have you had IVF?	(*n* = 975)	(*n* = 39)	0.139
Yes	266 (27.3%)	6 (15.4%)	
Have you had a pregnancy loss?	(*n* = 1,036)	(*n* = 43)	0.517
Yes	377 (36.4%)	13 (30.2%)	
Have you delayed having a family due to work?	(*n* = 973)	(*n* = 39)	0.407
Yes	591 (60.7%)	21 (53.8%)	
Seniority when you had first child	(*n* = 961)	(*n* = 38)	0.111
Intern	24 (2.5%)	2 (5.3%)	
Resident	106 (11.0%)	5 (13.2%)	
Registrar	454 (47.2%)	24 (63.2%)	
Fellow	116 (12.1%)	3 (7.9%)	
Consultant	261 (27.2%)	4 (10.5%)	
How many hours did you work per week?	(*n* = 970)		
<40	376 (38.8%)	–	
40–60	498 (51.3%)	–	
60–80	83 (8.6%)	–	
>80	13 (1.3%)	–	
Partner work hours		(*n* = 39)	0.023^**^
<40	–	26 (66.7%)	
40–60	–	13 (33.3%)	
Did you reduce your work schedule	(*n* = 971)		
Yes	263 (27.1%)	–	
Frequency of on call roster	(*n* = 972)		
None	393 (40.4%)	–	
2–4/month	323 (33.2%)	–	
4–6/month	126 (13.0%)	–	
>6/month	130 (13.4%)	–	
Shift length in the third trimester	(*n* = 968)		
0–8 h	251 (25.9%)	–	
8–12 h	592 (61.2%)	–	
12–16 h	82 (8.5%)	–	
>16 h	43 (4.4%)	–	
Any pregnancy complications	(*n* = 974)	(*n* = 39)	0.515
Yes	486 (49.9%)	17 (43.6%)	
NICU requirement	(*n* = 972)	(*n* = 39)	0.184
Yes	157 (16.2%)	3 (7.7%)	
Neonatal complication	(*n* = 966)	(*n* = 39)	0.564
Yes	228 (23.6%)	11 (28.2%)	

* Comparing age at first child's birth (female group) to Age of female partner at first child's birth (male group).

** Comparing Hours per week (female group) to Partner Work Hours (male group).

Further comparisons within the female participants were performed, looking at the three commonest specialty subgroups: surgeons, physicians, and general practitioners (Table 2). Despite surgeons being older at the time of their first child's birth, they were more likely to still be registrars, compared to general practitioners who were younger and more likely to be consultants. Over two thirds of surgeons and physicians stated they had delayed having a family due to work. There were significant differences in work schedules, with surgeons registering longer weekly hours, longer shifts and more on call. There were no differences in pregnancy-related complications. Although general practitioners had greater flexibility in returning to work, they also had less access to maternity leave.

**Table 2:** Comparison between surgical, medical, and general practice sub-specialities.

	**Surgery (*****n*** = **154)**	**Medicine (*****n*** = **169)**	**General practice (*n* = 287)**	***p*** **-value**
Age participant (mean ± sd)	37.6 ± 5.4	36.8 ± 4.3	36.4 ± 5.7	0.060
Age at first child's birth (median, IQR)	32 (31, 35)	33 (31, 35)	31 (29, 34)	<0.001
How many biological children?	(*n* = 131)	(*n* = 160)	(*n* = 272)	0.491
1	61 (46.6%)	77 (48.1%)	113 (41.5%)	
2	50 (38.2%)	67 (41.9%)	110 (40.4%)	
3	15 (11.5%)	15 (9.4%)	39 (14.3%)	
4	4 (3.1%)	1 (0.6%)	8 (2.9%)	
>4	1 (0.8%)	0	2 (0.7%)	
Have you had fertility testing?	(*n* = 154)	(*n* = 169)	(*n* = 286)	0.720
Yes	60 (39.0%)	64 (37.9%)	101 (35.3%)	
Have you had IVF?	(*n* = 131)	(*n* = 161)	(*n* = 274)	0.796
Yes	37 (28.2%)	40 (24.8%)	74 (27.0%)	
Total number of reproductive cycles (median, IQR)	4 (2, 5)	2 (1, 5)	3 (2, 6)	0.023
Have you had a pregnancy loss?	(*n* = 153)	(*n* = 169)	(*n* = 285)	0.971
Yes	56 (36.6%)	63 (37.3%)	103 (36.1%)	
Have you delayed having a family due to work?	(*n* = 131)	(*n* = 160)	(*n* = 274)	0.002
Yes	90 (68.7%)	108 (67.5%)	146 (53.3%)	
Seniority when you had first child	(*n* = 130)	(*n* = 160)	(*n* = 268)	<0.001
Intern	3 (2.3%)	2 (1.3%)	10 (3.7%)	
Resident	10 (7.7%)	11 (6.9%)	41 (15.3%)	
Registrar	76 (58.5%)	86 (53.8%)	79 (29.5%)	
Fellow	15 (11.5%)	20 (12.5%)	50 (18.7%)	
Consultant	26 (20.0%)	41 (25.6%)	88 (32.8%)	
How many hours did you work per week?	(*n* = 131)	(*n* = 159)	(*n* = 273)	<0.001
<40	16 (12.2%)	42 (26.4%)	174 (63.7%)	
40–60	62 (47.3%)	107 (67.3%)	90 (33.0%)	
60–80	44 (33.6%)	10 (6.3%)	7 (2.6%)	
>80	9 (6.9%)	0	2 (0.7%)	
Did you reduce your work schedule	(*n* = 131)	(*n* = 159)	(*n* = 274)	<0.001
Yes	32 (24.4%)	35 (22.0%)	110 (40.1%)	
Shift length in the third trimester	(*n* = 131)	(*n* = 158)	(*n* = 274)	<0.001
0–8 h	21 (16.0%)	33 (20.9%)	118 (43.1%)	
8–12 h	67 (51.1%)	110 (69.6%)	131 (47.8%)	
12–16 h	35 (26.7%)	10 (6.3%)	9 (3.3%)	
>16 h	8 (6.1%)	5 (3.2%)	16 (5.8%)	
Any pregnancy complications	(*n* = 131)	(*n* = 161)	(*n* = 273)	0.968
Yes	68 (51.9%)	82 (50.9%)	138 (50.5%)	
Complications leading to time off work	(*n* = 67)	(*n* = 81)	(*n* = 138)	0.897
Yes	42 (62.7%)	53 (65.4%)	91 (65.9%)	
Colleague supportive of extra time off	(*n* = 42)	(*n* = 53)	(*n* = 91)	0.001
Yes	25 (59.5%)	45 (84.9%)	78 (85.7%)	
NICU requirement	(*n* = 130)	(*n* = 161)	(*n* = 273)	0.866
Yes	25 (19.2%)	30 (18.6%)	47 (17.2%)	
Neonatal complication	(*n* = 131)	(*n* = 160)	(*n* = 271)	0.887
Yes	31 (23.7%)	37 (23.1%)	68 (25.1%)	
Recommend medical career to child?	(*n* = 153)	(*n* = 166)	(*n* = 284)	0.148
Yes	71 (46.4%)	82 (49.4%)	158 (55.6%)	
Access to maternity leave	(*n* = 154)	(*n* = 168)	(*n* = 266)	<0.001
Yes	119 (77.3%)	138 (82.1%)	85 (32.0%)	
Length of maternity leave in weeks (median, IQR)	20 (10, 26)	28 (16, 40)	26 (18, 48)	<0.001
Return to work	(*n* = 131)	(*n* = 160)	(*n* = 274)	<0.001
Full time	87 (66.4%)	61 (38.1%)	32 (11.7%)	
Part time	44 (33.6%)	98 (61.3%)	235 (85.8%)	
Did not return	0	1 (0.6%)	7 (2.6%)	

When performing an analysis of women who had recorded a pregnancy-related complication, a higher proportion had undergone fertility testing, IVF and had suffered a previous pregnancy loss (Table 3). They were also more likely to report neonatal complications. Factors such as age at first child's birth or shift length in the third trimester were not different between groups. The presence of birth complications was associated with a lower likelihood of successful initiation of breastfeeding (78.6 vs. 85.2%, *p* = 0.023).

**Table 3:** Factors associated with pregnancy complication.

	**Complication**	**No complication**	***p*** **-value**
Age at first child's birth (median, IQR)	32 (30, 34)	32 (30, 34)	0.210
Have you had fertility testing?	(*n* = 486)	(*n* = 487)	<0.001
Yes	212 (43.6%)	149 (30.6%)	
Have you had IVF?	(*n* = 486)	(*n* = 488)	<0.001
Yes	162 (33.3%)	104 (21.3%)	
Total number of reproductive cycles (median, IQR)	3 (1, 5)	2 (1, 5)	0.369
Have you had a pregnancy loss?	(*n* = 215)	(*n* = 139)	<0.001
Yes	215 (44.3%)	139 (28.6%)	
Have you delayed having a family due to work?	(*n* = 486)	(*n* = 486)	<0.001
Yes	314 (64.6%)	277 (57.0%)	
Did you reduce your Work Schedule	(*n* = 484)	(*n* = 486)	<0.001
Yes	159 (32.9%)	103 (21.2%)	
Shift length in the third trimester	(*n* = 481)	(*n* = 486)	0.925
0–8 h	129 (26.8%)	122 (25.1%)	
8–12 h	292 (60.7%)	299 (61.5%)	
12–16 h	39 (8.1%)	43 (8.8%)	
>16 h	21 (4.4%)	22 (4.5%)	
NICU requirement	(*n* = 485)	(*n* = 486)	<0.001
Yes	127 (26.2%)	30 (6.2%)	
Neonatal complication	(*n* = 481)	(*n* = 484)	<0.001
Yes	179 (37.2%)	49 (10.1%)	
Wellbeing this week—scale 1–10 (median, IQR)	7 (5, 7)	7 (6, 8)	<0.001

87.9% of female participants had initiated breastfeeding successfully, with 81.9% of those breastfeeding exclusively (Table 4). Only 67.8% could express whilst working, with finding a suitable location to express only found “often” by 20.4% of those breastfeeding. Colleagues were deemed usually or always supportive by only 56.6% of respondents. However, 68.9% of women continued breastfeeding at least 6 months and only 24.9% indicated that cessation was due to demands at work.

**Table 4:** Breastfeeding practices among ANZ doctors.

Infant feeding method at birth	(*n* = 972)
Exclusive breastfeeding	796 (81.9%)
Combination breast mild and formula	156 (16.0%)
Not breastfeeding at all	20 (2.1%)
Emotional state during breastfeeding	(*n* = 949)
Severely depressed	42 (4.4%)
Mildly depressed	320 (33.7%)
Not depressed at all	587 (61.9%)
Express while working	(*n* = 918)
Yes	622 (67.8%)
Sufficient time to express at work	(*n* = 619)
Yes	288 (46.5%)
Access to appropriate place to express at work	(*n* = 619)
Never	113 (18.3%)
Occasionally	121 (19.5%)
Sometimes	127 (20.5%)
Often	126 (20.4%)
Always	132 (21.3%)
Were colleagues supportive of milk expression at work?	(*n* = 618)
Always opposed	1 (0.2%)
Usually opposed	18 (2.9%)
Neither supportive nor oppositional	161 (26.1%)
Usually supportive	198 (32.0%)
Always supportive	152 (24.6%)
Colleagues did not know	76 (12.3%)
Not applicable	12 (1.9%)
Duration of breast feeding	(*n* = 910)
<3 months	109 (12.0%)
3–6 months	174 (19.1%)
6–12 months	279 (30.7%)
>12 months	348 (38.2%)
Was discontinuation of breastfeeding due to demands at work?	(*n* = 906)
Yes	226 (24.9%)
No	602 (66.4%)
Still breastfeeding	78 (8.6%)
Satisfied with duration of breastfeeding?	(*n* = 912)
Yes	684 (75.0%)
No	164 (18.0%)
Still breastfeeding	64 (7.0%)

Breastfeeding practices varied across the different sub-specialities. Surgeons experienced the greatest barriers to flexible rostering to allow the continuation of breastfeeding. Insufficient time to express at work was a common issue raised in our study, and one in four female doctors never had an appropriate place to express at work (Table 5). Overall, the majority (68.9%) of the respondents in this study breastfed more than 6 months, and the majority (75%) were satisfied with their breastfeeding efforts. Workplace demands resulted in discontinuation of breastfeeding in the majority of surgeons (48.7% surgeons vs. 20.5% physicians and 12.6% general practitioners, *p* < 0.001), and an overall decreased rate of satisfaction with the duration of breastfeeding.

**Table 5:** Breastfeeding practices comparison among sub-specialties.

	**Surgery (*n* = 154)**	**Medicine (*n* = 169)**	**General Practice (*n* = 287)**	***p*-value**
Infant feeding method at birth	(*n* = 131)	(*n* = 161)	(*n* = 273)	0.160
Exclusive breastfeeding	101 (77.1%)	131 (81.4%)	228 (83.5%)	
Combination breast mild and formula	23 (17.6%)	27 (16.8%)	41 (15.0%)	
Not breastfeeding at all	7 (5.3%)	3 (1.9%)	4 (1.5%)	
Emotional state during breastfeeding	(*n* = 124)	(*n* = 157)	(*n* = 269)	0.079
Severely depressed	10 (8.1%)	7 (4.5%)	10 (3.7%)	
Mildly depressed	44 (35.5%)	66 (42.0%)	86 (32.0%)	
Not depressed at all	70 (56.5%)	84 (53.5%)	173 (64.3%)	
Express while working	(*n* = 119)	(*n* = 153)	(*n* = 259)	0.048
Yes	91 (76.5%)	97 (63.4%)	169 (65.3%)	
Sufficient time to express at work	(*n* = 91)	(*n* = 96)	(*n* = 169)	0.002
Yes	32 (35.2%)	40 (41.7%)	96 (56.8%)	
Access to appropriate place to express at work	(*n* = 91)	(*n* = 96)	(*n* = 168)	<0.001
Never	30 (33.3%)	17 (17.7%)	15 (8.9%)	
Occasionally	17 (18.7%)	20 (20.8%)	23 (13.7%)	
Sometimes	22 (24.2%)	30 (31.3%)	22 (13.1%)	
Often	10 (11.0%)	22 (22.9%)	32 (19.0%)	
Always	12 (13.2%)	7 (7.3%)	76 (45.2%)	
Were colleagues supportive of milk expression at work?	(*n* = 90)	(*n* = 96)	(*n* = 168)	0.002
Always opposed	0	0	1 (0.6%)	
Usually opposed	8 (8.9%)	1 (1.0%)	3 (1.8%)	
Neither supportive nor oppositional	32 (35.6%)	26 (27.1%)	38 (22.6%)	
Usually supportive	24 (26.7%)	32 (33.3%)	39 (23.2%)	
Always supportive	17 (18.9%)	18 (18.8%)	26 (15.5%)	
Colleagues did not know	9 (10.0%)	18 (18.8%)	26 (15.5%)	
Not applicable	0	1 (1.0%)	5 (3.0%)	
Duration of breast feeding	(*n* = 119)	(*n* = 153)	(*n* = 255)	0.070
<3 months	17 (14.3%)	19 (12.4%)	24 (9.4%)	
3–6 months	29 (24.4%)	29 (19.0%)	45 (17.6%)	
6–12 months	39 (32.8%)	53 (34.6%)	72 (28.2%)	
>12 months	34 (28.6%)	52 (34.0%)	114 (44.7%)	
Was discontinuation of breastfeeding due to demands at work?	(*n* = 119)	(*n* = 151)	(*n* = 253)	<0.001
Yes	58 (48.7%)	31 (20.5%)	32 (12.6%)	
No	48 (40.3%)	107 (70.9%)	197 (77.9%)	
Still breastfeeding	13 (10.9%)	13 (8.6%)	24 (9.5%)	
Satisfied with duration of breastfeeding?	(*n* = 119)	(*n* = 152)	(*n* = 256)	0.001
Yes	76 (63.9%)	110 (72.4%)	207 (80.9%)	
No	33 (27.2%)	32 (21.1%)	27 (10.5%)	
Still breastfeeding	10 (8.4%)	10 (6.6%)	22 (8.6%)	

## Discussion

The results of this study suggest that female doctors may experience many obstacles in family planning. The strenuous training requirements for many young doctors possibly contribute to these difficulties. Thus, as more women enter medicine and its sub-specialities, and as the age of childbearing advances, it becomes increasingly important to address the culture and issues surrounding parenthood in the medical workforce.

When considering the optimal time to start a family, the perceived negative impacts of childbearing on a woman's career may often be at the forefront of her mind. This may delay starting a family until later in life, with participants in this study recording an average age of first-time mothers of 32.4 years. Although a direct comparison is difficult, due to potential differences in the studied cohort, the Australian national average is 29.4 (9). Surgeons, in particular, were found to be the most likely to postpone pregnancy due to training commitments, compared to other medical backgrounds. Several studies have now highlighted the adverse effects of advancing maternal age, with its increased risks of infertility and adverse pregnancy outcomes (4). This study results support these findings, as one in three women reported having a miscarriage, compared to one in five with the general population (10). Despite the known escalating risk of infertility with advancing maternal age, more than half of doctors in this study's cohort delayed starting their family due to work requirements. The perceived expectation that young women should wait until the end of training to have children may account for the high rate of respondents who needed fertility testing (37%). Moreover, a substantial portion of them went on to require ART, such as IVF (27.3%). The length of medical training, as much as 14 years in some sub-specialties, may well contribute to the older maternal age, and may necessitate a disproportionate number of doctors having to utilize ART.

Regarding the practicality of ART, the considerable expense, frequent appointments, repeated procedures and laboratory tests may become a large burden to already time-poor doctors. It is not surprising that those who require fertility testing and ART are more likely to have worse mental health outcomes (11). Additionally, ART, particularly IVF, is known to contribute to worse pregnancy and neonatal outcomes, which may carry long-term implications for the mental and physical wellbeing of both mother and child (12). For these compelling reasons, training colleges and health services need to work together to implement the necessary strategies and infrastructure for their trainees to have families during training. These practical measures appear to be necessities, but equally important is the need for cultural change. In a largely male dominated profession where full time lengthy training programs are the norm, acceptance of breaks in training, part time training, shortened days and prolonged rest times needs to be commonplace. If registrars feel supported enough to not delay childbearing until the end of a lengthy training program, their younger maternal age may reduce the rates of infertility and need for ART (6).

Pregnancy complications rates for doctors have been found to be between 34 and 48.3% (4), which is consistent with the findings of this study in which half of the surveyed medical mothers experienced a pregnancy complication. Moreover, these pregnancy complications were also more likely to necessitate the need for NICU admissions for their newborns. To explain the potential correlation between the medical profession and reproductive disorders, studies have put forward several potential causative factors. Takeuchi et al. found that long working hours may be hazardous to fetal health, with a three-fold increased risk of experiencing threated abortion (13). Eighty five percentage of the surveyed medical mothers in this study were still working 8–12+ h per shift during their third trimester. Other possible explanations include regular exposure to occupational hazards to doctors at work, such as ionizing radiation, electromagnetic fields, communicable diseases, cytotoxic and other chemical agents, surgical smoke, and physical stress (such heavy lifting, stair climbing, or night shifts) (14, 15). Whilst mothers-to-be will do their best to minimize contact with these exposures, the nature of medical work often makes complete isolation from them impossible.

The World Health Organization strongly advocates for breastfeeding to be an important component of childrearing, as it offers benefits to both the mother and her newborn baby (16). Whilst the right to breastfeeding in public is enshrined in Australian law, the requirements on employers are less clear and are covered by state laws. The Victorian law, for example, says that employers must “reasonably accommodate” employees who wish to continue breastfeeding. Key components of a successful breastfeeding include adequate parental leave (both maternity and paternity), safe space to express, adequate time to express milk at work, and supportive environment for continuation in milk supply (17). However, surgeons as a subgroup were most susceptible to early cessation of breastfeeding, possibly due to the unyielding demands of surgical work. Surgical respondents, of the specialties analyzed, ranked highest for insufficient time and lack of designated private spaces for milk expression. Lack of flexibility in returning to work in a part-time arrangement may further compromise the breastfeeding efforts of surgeon mothers. These workplace limitations may have resulted in earlier discontinuation of breastfeeding in the majority of surgeons compared with their colleagues (48.7% surgeons vs. 20.5% physicians vs. 12.6% GPs *p* < 0.001). These trends are reflected in literature, as authors Sattari et al. showed 43% doctor mothers terminated breastfeeding early due to demands of work (17).

Systemic factors within hospital rostering and infrastructure need to be addressed to reduce the inadequate time and space for working mothers to breastfeed or express. This study's findings suggest that there is a need for simple structural strategies to be implemented, such as setting aside a room for expressing, furnishing the space with a fridge for storing expressed breast milk and allocating a break between clinic patients and operating lists. Awareness of the benefits of a pregnancy-friendly and lactation-friendly workplace is wise. These include retaining highly-skilled staff, reducing absenteeism, and fostering a healthy working culture in the medical work place (18), as well as improved physical and mental wellbeing of the medical mothers and infants, and achievement of their lactation goals.

To address the inflexibility during training, part-time training positions should be incorporated into the training structure to minimize delay in starting a family and be part of accepted as part of a culture change across the profession. Access to parental leave (both males and females) should be prioritized to meet the demands of the rising number of female doctors. Safe access and adequate time to breastfeed or express is not only a legal requirement, but also a fundamental requirement to return to work for working mothers and should be an institution's performance indicator. Crucially, a focus on education around infertility and pregnancy complications, as well as collegiate support through a culture change should be implemented by training bodies.

## Limitations

Due to the recruitment and advertising process, it is not clear whether the results of this study are representative of the Australian and New Zealand medical community. Due to the voluntary participation, there is a considerable risk of selection bias as medical mothers experiencing childbearing issues are more likely to seek support on the Facebook Group and more likely to be involved in such a survey. There was a very low male participation rate, and given the likely participation bias by affected males in the Facebook Group, this is likely to bias the gender comparisons.

## Conclusion

This study underscores the considerably high rates of infertility and pregnancy complications amongst a cohort of Australian and New Zealand medical professionals. Delays in starting a family due to work-related factors, advancing maternal age, and long irregular working hours may contribute to this incidence. Structural barriers to breastfeeding, in the hospital or clinic environment, could impair the efforts of working mothers to continue breastfeeding beyond 6 months. Interventions to provide improved working conditions for doctors are urgently needed to improve the overall risks of pregnancy and childbearing, and warrants further research. Professional training institutions and employers need to address this issue in a proactive and expedient manner.

## Data availability statement

The original contributions presented in the study are included in the article/supplementary material, further inquiries can be directed to the corresponding author.

## Ethics statement

The studies involving human participants were reviewed and approved by Northern Health Ethics and Governance Committee (HREC 79540). The patients/participants provided their written informed consent to participate in this study.

## Author contributions

JK, RH, and GC: conception and design, development of methodology, acquisition of data (provided animals, acquired and managed patients, provided facilities, etc.), and analysis and interpretation of data (e.g., statistical analysis, biostatistics, computational analysis). JK, KS, RH, and GC: writing, review, and/or revision of the manuscript and administrative, technical, or material support (i.e., reporting or organizing data, constructing databases). RH and GC: study supervision. All authors contributed to the article and approved the submitted version.

## Funding

REDCap development was supported by NIH/NCATS Colorado CTSA Grant Number UL1 TR002535.

## Conflict of interest

The authors declare that the research was conducted in the absence of any commercial or financial relationships that could be construed as a potential conflict of interest.

## Publisher's note

All claims expressed in this article are solely those of the authors and do not necessarily represent those of their affiliated organizations, or those of the publisher, the editors and the reviewers. Any product that may be evaluated in this article, or claim that may be made by its manufacturer, is not guaranteed or endorsed by the publisher.

## Author disclaimer

The publication's contents are the authors' sole responsibility and do not necessarily represent official NIH views.
